# Suv39h1 promotes facet joint chondrocyte proliferation by targeting miR-15a/Bcl2 in idiopathic scoliosis patients

**DOI:** 10.1186/s13148-019-0706-1

**Published:** 2019-07-23

**Authors:** Jiong Li, Guanteng Yang, Shaohua Liu, Longjie Wang, Zhuotao Liang, Hongqi Zhang

**Affiliations:** Department of Spine Surgery, Xiangya Hospital, Central South University, No. 87, Xiangya Road, Changsha, 410008 China

**Keywords:** Idiopathic scoliosis, Facet joint, Chondrocyte proliferation, Histone methylation, SUV39H1, miR-15a/Bcl2

## Abstract

**Background:**

Idiopathic scoliosis (IS) is a complex disease with an unclear etiology, and the worldwide prevalence is approximately 2–3%. As an important link between environmental factors and phenotypic differences, epigenetic changes, such as lncRNA, miRNA, and DNA methylation, have recently been reported to be associated with the development of IS. However, the correlation between histone methylation, another classical epigenetic mechanism, and IS has not been determined. In this study, we investigated the morphological changes, alterations in the levels of histone methylation, and cell proliferation-related pathway in inferior facet joint cartilage in 11 IS patients and 10 comparable controls.

**Results:**

Compared with the control group, narrowed facet joint cartilage but increased proliferative chondrocytes and upregulated collagen type II (*COL2A1*) and B-cell lymphoma-2 (Bcl2) were observed in IS patients. Additionally, tri-methylation levels of H3K9 (H3K9me3) rather than other lysine sites were significantly increased in IS patients, coinciding with the upregulation of its specific enzyme, suppressor of variegation 3-9, drosophila homolog of 1 (SUV39H1). In addition, Bcl2-targeted miR-15a was downregulated in IS patients, and the level of H3K9me3 in the promoter region of the miR-15a host gene was remarkably increased in IS patients compared with the control group. Moreover, overexpressing SUV39H1 in ATDC5 cells with increased H3K9me3 levels led to similar changes, with increased expression of COL2A1 and Bcl2, decreased expression of miR-15a, and increased cell proliferation.

**Conclusions:**

Thus, our study suggests that increased chondrocyte proliferation occurs in the facet joint cartilage of IS patients compared with the control group and may be promoted by the elevated levels of H3K9me3 and SUV39H1, which regulate the miR-15a/Bcl2 pathway. This dysregulation of chondrocyte proliferation could result in abnormal spinal growth and may additionally participate in the development and progression of IS.

**Electronic supplementary material:**

The online version of this article (10.1186/s13148-019-0706-1) contains supplementary material, which is available to authorized users.

## Background

Idiopathic scoliosis (IS) is a three-dimensional spinal deformity disease that mostly occurs during adolescence (adolescent idiopathic scoliosis, AIS). The worldwide prevalence of AIS ranges from 0.47–5.2% [1]. Although the exact etiology of IS remains unknown, several theories related to the development of IS have been established, including genetics, endocrine metabolism, biochemics, biomechanics, and environmental factors [2].

Some studies have suggested that the fundamental problem in scoliosis is the imbalanced growth between the anterior and posterior components of the spine, manifested by longer anterior elements of the spine than the posterior elements in IS patients [3, 4]. Moreover, high spinal growth velocity is predisposed to a more rapid curve progression [5]. In addition to growth-related factors, joint hypermobility may be susceptible to spinal instability and the development of IS, since researchers have found that girls with IS have significantly more laxity than that of healthy sex- and age-matched controls [6, 7]. Pal et al. found that scoliosis developed rapidly and was considerable after both transverse processes and facet joints were removed [8]. Therefore, abnormal growth of the vertebral column indeed may be tightly associated with the development of IS.

Discordant phenotypes for monozygotic twins with AIS suggested that environmental factors are important to the development of IS, although what kind of environmental factors may be involved is unclear [9]. The influence of environmental factors can be linked to phenotypic differences by epigenetic changes, mechanisms of which mainly contain DNA methylation, histone modification, and noncoding RNAs [10, 11]. Epigenetic changes can regulate phenotypic plasticity with complex regulation during the early stage of life but are also involved in the development of many diseases, such as cancer, diabetes, cardiovascular disease, and liver disease [9, 10, 12, 13].

Similarly, changes in epigenetics were also found in AIS patients compared with the control group. A total of 139 long noncoding RNAs (lncRNAs) were differentially expressed between AIS patients and healthy controls, and 4 lncRNAs were expressed differently between different patients when grouped according to age, height, classification, severity of scoliosis, and Risser grade [14]. Garcia-Gimenez et al. observed several deregulated microRNAs (miRNAs) that participate in the differentiation mechanisms of osteoblasts/osteoclasts in AIS patients [15]. Ogura et al. found that the risk allele of a functional variant in MIR4300HG, the host gene of miR4300, can lead to a decrease in miR4300 and be related to AIS progression [16]. In addition, AIS patients had significantly higher *COMP* (cartilage oligomeric matrix protein) promoter methylation and lower gene expression compared with those of the control groups, which was correlated with young age and a high Cobb angle of the main curve [17]. Although strong associations between rs12459350 in Disruptor of telomeric silencing 1-like (*DOT1L*) that catalyze methylation of histone H3 lysine 79 (H3K79) and susceptibility of AIS were found [18], the correlation between alteration of histone methylation and development of IS has not been discovered. In this study, we examined histone methylation levels in facet joint chondrocytes, which are important to spinal growth, in IS patients and the control group, and further investigation between changes in histone methylation and the development of IS was conducted.

## Results

### General characteristics of IS patients and control groups

Overall, a population of 11 IS patients and 10 comparable controls were investigated in our study. The mean age of IS patients was 16.86 years, ranging from 15 to 21 years, while the mean age of the control group was 19.80 years, ranging from 16 to 28 years (Table 1 and Additional file 1: Table S1). However, there was no significant difference between the ages of the two groups (Table 1). Moreover, the gender difference between the two groups was not observed, since there were 6 males/4 females in the control group and 5 males/6 females in the IS patient group (Table 1). Additionally, the average Cobb angle of the major curve in IS patients was 45.59° (Table 1).

**Table 1:** Demographic of study populations

Parameters	CT *N* = 10	IS *N* = 11	*p* value
Age (years)	19.80 ± 4.57	16.86 ± 1.86	0.0641
Gender	M 6 / F 4	M 5 / F 6	0.410
Major curve Cobb angle (°)	–	45.59 ± 16.38	–

*CT* control group, *IS* idiopathic scoliosis patients, *F* female, *M* male. Data was present as mean ± standard deviation

### Significant changes in the cartilage layer of the facet joint in IS patients

**Fig. 1:**
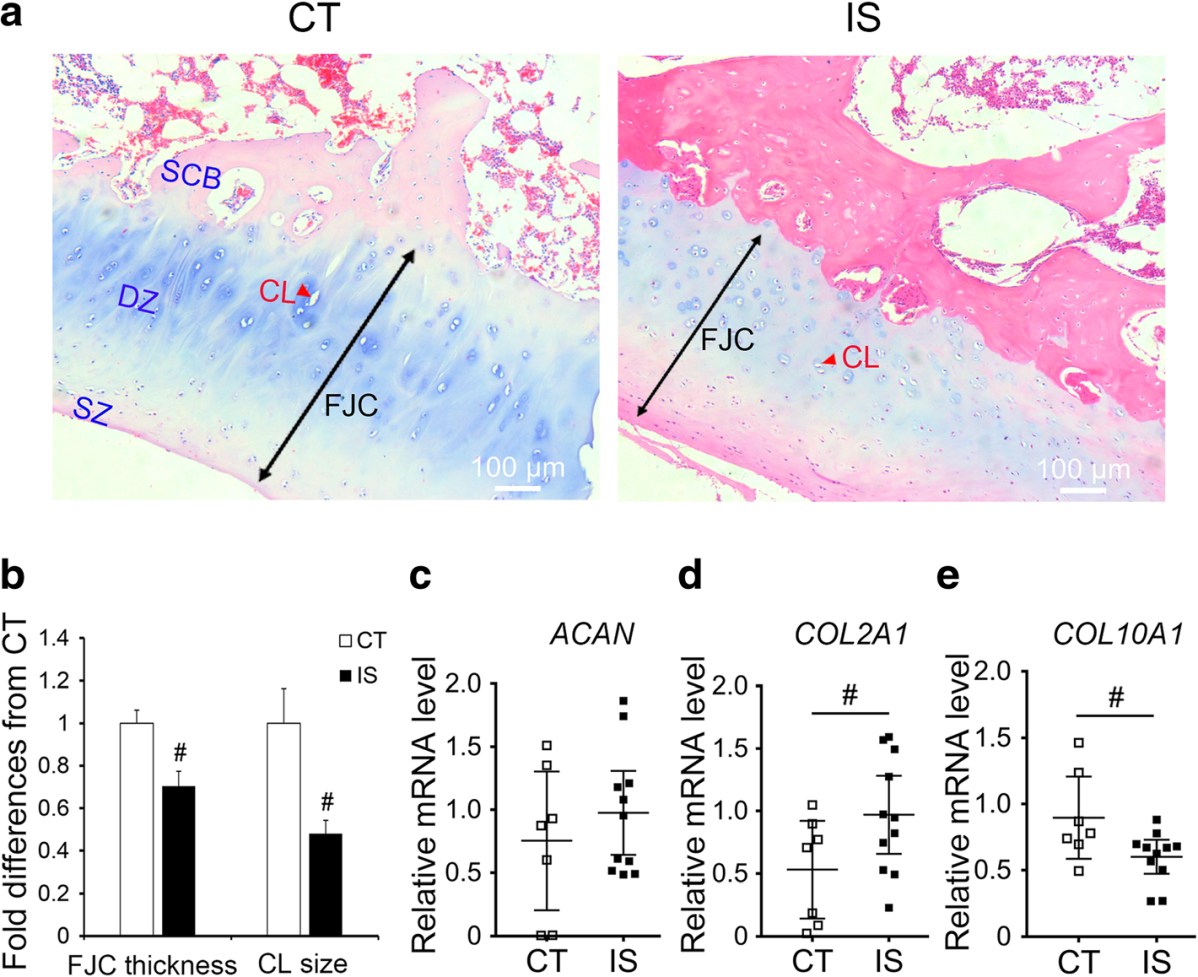
Histopathological analysis of facet joint cartilage in IS patients and control group. **a** H&E stained facet joint sections of IS patients and control group (× 100). Black double arrow indicates FJC. Red arrow indicates CL. **b** Quantity analysis of facet joint cartilage in IS patients and control group. **c**–**e** the mRNA levels of chondrocytes extracellular matrix-related genes. FJC, facet joint cartilage; CL, cartilage lacuna; SCB, subchondral bone; DZ, deep zone; SZ, superficial zone. CT, *n* = 10; IS patients, *n* = 11, ^#^*p* < 0.05

The histopathological features of facet joint cartilage in IS patients and the control group were analyzed; however, severely damaged facet joints of IS patients that lacked an obvious cartilage layer were excluded from the analysis. In general, articular cartilage has a highly organized structure composed of 4 zones: the superficial zone, middle zone, deep zone, and calcified zone [19]. The chondrocytes in the superficial region are smaller and flatter when compared to the middle and deep zones where cells are organized in a more columnar-shaped cartilage lacuna [20]. As depicted in Fig. 1 a, hematoxylin-eosin (H&E) staining results suggested that the surface, middle, and deep zones of the cartilage were well preserved in both the control group and IS patients. However, a significantly narrowed cartilage layer of the facet joint was observed in IS patients compared with the control group (Fig. 1a, b). In addition, changes in the chondrocyte phenotype in the middle and deep zones of cartilage were observed between the two groups, showing increased smaller and fewer columnar chondrocytes in IS patients compared with the control group (Fig. 1a, b).

Furthermore, we examined the mRNA levels of extracted primary chondrocyte extracellular matrix components aggrecan (*ACAN*), collagen type II (*COL2A1*), and collagen type X (*COL10A1*), which is a reliable marker for new bone formation in articular cartilage [21, 22]. As shown in Fig. 1 d and e, the mRNA level of *COL2A1* was significantly increased in IS patients compared with the control group, whereas *COL10A1* markedly decreased. There was no significant change in *ACAN* mRNA levels between the two groups (Fig. 1c).

### Alteration in histone methylation in the facet joint chondrocytes of IS patients compared with the control group

**Fig. 2:**
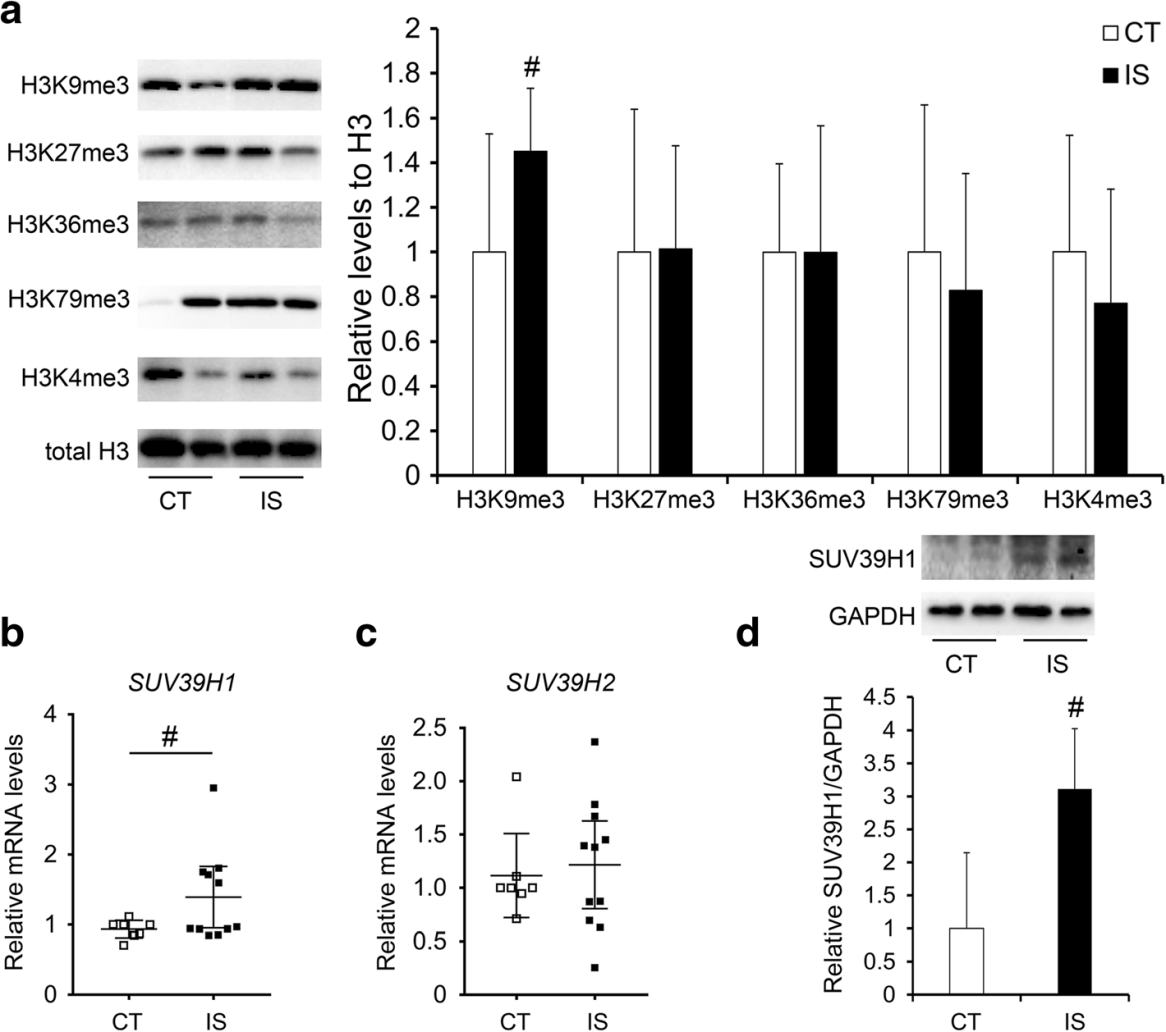
Alteration of histone methylation in the facet joint chondrocytes of IS patients. **a** tri-methylation levels of different H3 lysine sites in the facet joint chondrocytes of IS patients and control group. **b**–**c** the mRNA levels of H3K9me3 specific methyltransferases in IS patients and control group. **d** the protein levels of SUV39H1 in IS patients and control group. CT, *n* = 7; IS patients, *n* = 11, ^#^*p* < 0.05

Given that histone methylation plays important roles in chondrocyte proliferation and hypertrophy [23], we detected the histone methylation levels of facet joint chondrocytes in both IS patients and the control group. According to the WB results, a significant increase in the H3K9me3 level, which is a marker of gene repression, was found in IS patients compared with the control group (Fig. 2a) [24]. However, another marker of gene repression, H3K27me3, was not changed in IS patients compared with the control group (Fig. 2a) [24]. Surprisingly, the markers of gene activation, such as H3K4me3, H3K36me3, and H3K79me3, did not change between the two groups (Fig. 2a) [24]. Next, we investigated the expression levels of H3K9 tri-methyltransferases, including suppressor of variegation 3-9, drosophila homolog of 1 and 2 (SUV39H1, SUV39H2) [23]. Although no changes in SUV39H2 expression were observed, the mRNA and protein levels of SUV39H1 were increased in IS patients compared with the control group (Fig. 2b–d). Meanwhile, no significant changes of the mRNA levels of another H3K9 methyltransferase SET domain bifurcated-1 (SETDB1) and H3K9 demethylases lysine (K)-specific demethylases 4 (KDM4A, KDM4B, and KDM4C) were observed between the two groups (Additional file 2: Figure S1).

### Increased proliferation of chondrocytes in the facet joint cartilage of IS patients

**Fig. 3:**
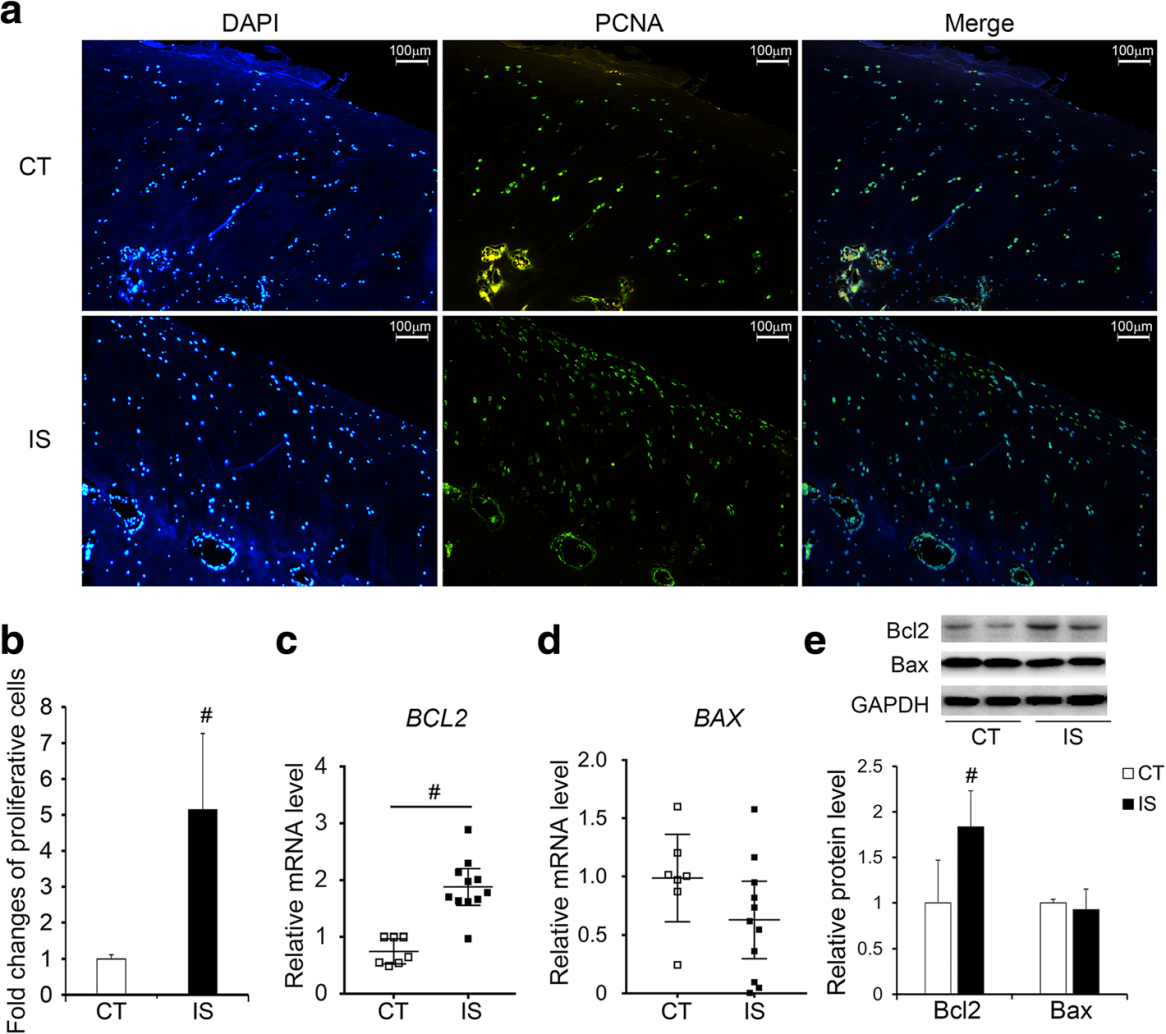
Increased proliferation of facet joint chondrocytes in IS patients. **a** Immunofluorescent stained PCNA in the facet joint sections of IS patients and control group (× 200). CT, *n* = 10; IS patients, *n* = 11. **b** Quantity evaluation of proliferative chondrocytes in IS patients and control group. CT, *n* = 10; IS patients, *n* = 11. **c**–**e** The mRNA and protein levels of Bcl2 and Bax in the facet joint chondrocytes of IS patients and control group. CT, *n* = 7; IS patients, *n* = 11. ^#^*p* < 0.05

We further measured chondrocyte proliferation in the facet joint cartilage of IS patients and the control group. In accordance with the H&E staining results, the immunofluorescent staining of proliferating cell nuclear antigen (PCNA), a marker for proliferation, suggested markedly increased proliferative cells of the facet joint chondrocytes in IS patients compared with the control group (Fig. 3a, b). Moreover, the expression of the anti-apoptosis protein B-cell lymphoma-2 (Bcl2) was increased in both the mRNA and protein levels of chondrocytes in IS patients, but no significant changes in either the mRNA or protein level of the pro-apoptosis protein Bcl2-associated x (Bax) were found in IS patients compared with the control group (Fig. 3c–e).

These data showed that the increased smaller chondrocytes we observed in IS patients may result from the enhanced proliferation of facet joint cartilage, which could be further induced by the increased expression of Bcl2. However, whether these changes were related to the alteration in H3K9me3 levels remains unclear, especially the gene inactivation role of H3K9me3 modification.

### H3K9me3 modification caused a decrease in miRNAs targeting Bcl2 in the facet joint chondrocytes of IS patients

**Fig. 4:**
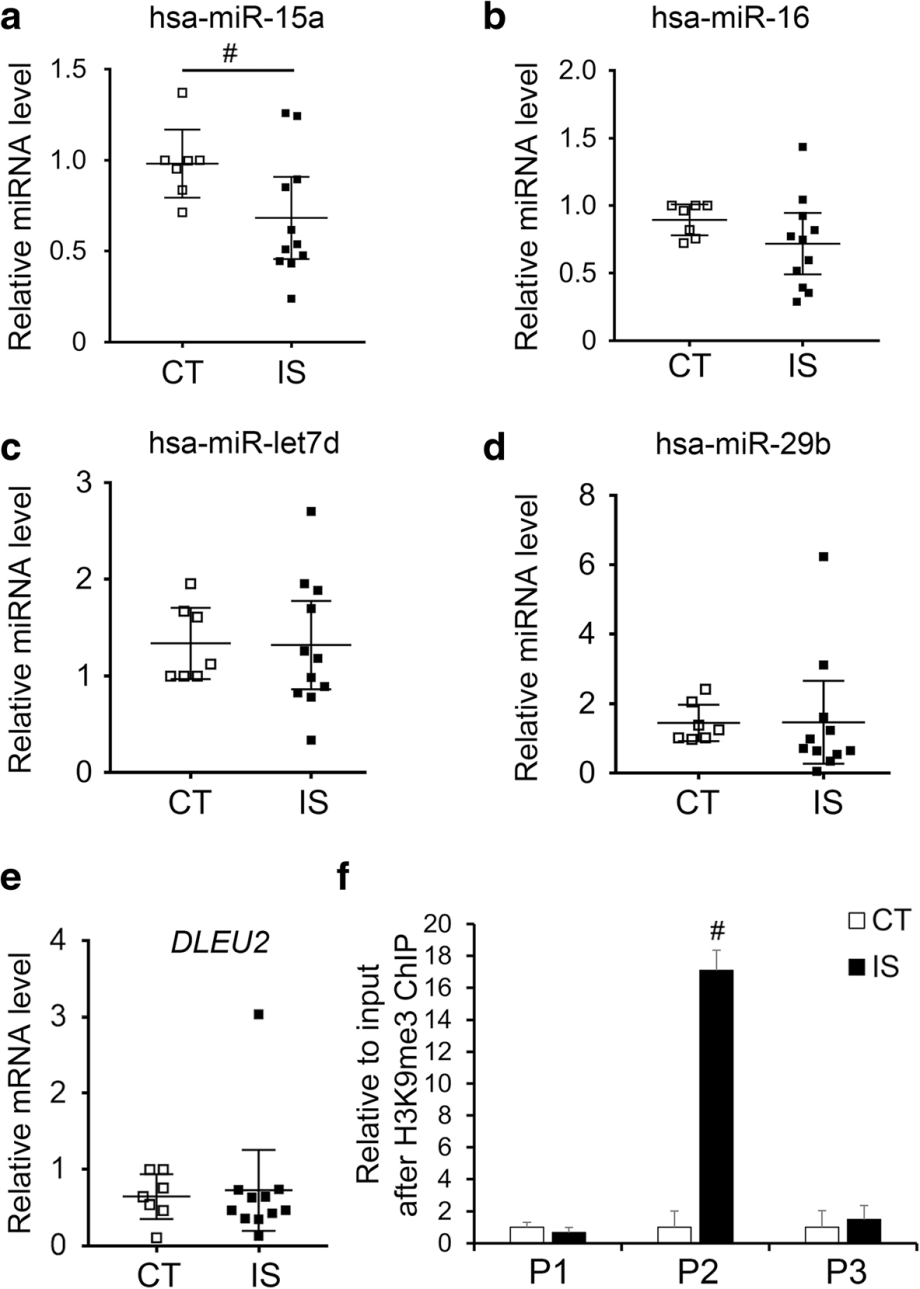
Changes of miRNAs targeting Bcl2 in the facet joint chondrocytes of IS patients. **a**–**d** The expression levels of miRNAs in the facet joint chondrocytes of IS patients and control group. CT, *n* = 7; IS patients, *n* = 11. **e** The mRNA level of miR-15a/16 host gene in the facet joint chondrocytes of IS patients and control group. CT, *n* = 7; IS patients, *n* = 11. **f** The levels of H3K9me3 in the promoter region of miR-15a/16 host gene in the facet joint chondrocytes of IS patients and control group assessed by ChIP assay. P1–P3, three pairs of primers in the promoter region of miR-15a/16 host gene. *n* = 3. ^#^*p* < 0.05

Based on the hypothesis that elevated Bcl2 expression may be caused indirectly by H3K9me3 modification, we searched for genes that downregulated the expression of Bcl2. By using miRTarBase (http://mirtarbase.mbc.nctu.edu.tw/php/search.php) and a review of the literature, we found four miRNAs that inhibit Bcl2 expression: the microRNA cluster miR-15a/miR-16, miR-29b, and miR-let7d [25–29]. As shown in Fig. 4 a–d, the expression level of hsa-miR-15a was significantly decreased in IS patients compared with the control group, while hsa-miR-16, hsa-miR-29b, and hsa-miR-let7d did not significantly change between the two groups. The analysis of the miR-15a/miR-16 host gene showed no changes in deleted in lymphocytic leukemia 2 (*DLEU2*) mRNA levels between IS patients and the control group (Fig. 4e) [30]. We further tested the association between the downregulation of has-miR-15a and elevation in H3K9me3 levels by chromatin immunoprecipitation (ChIP) assay. In accordance with our hypothesis, the H3K9me3 level was significantly increased in the promoter region of *DLEU2* (Fig. 4f and Additional file 2: Figure S2).

### Changes caused by overexpressing SUV39H1 in ATDC5 cells

**Fig. 5:**
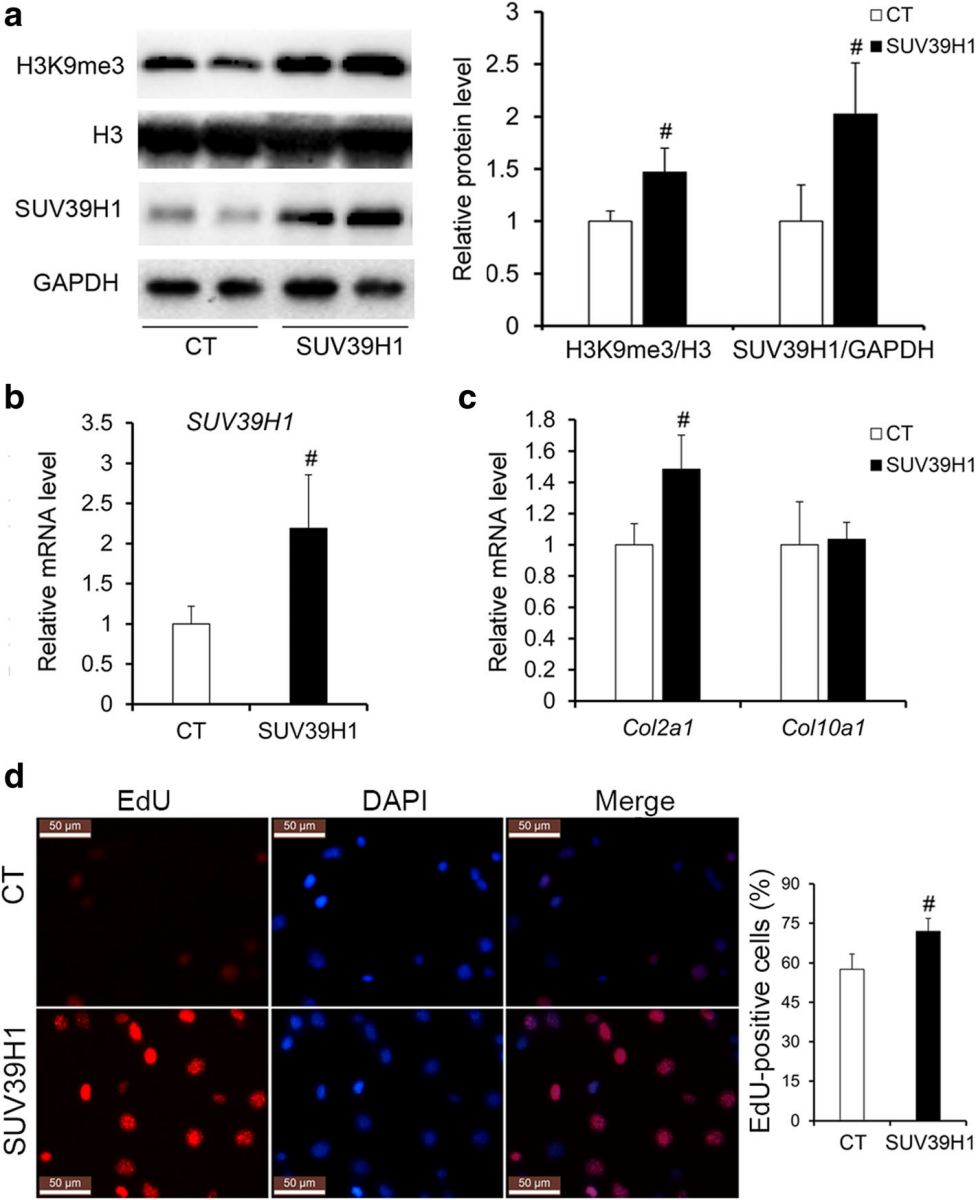
Overexpression of SUV39H1 in ATDC5 cells. **a** The protein levels of H3K9me3 and SUV39H1 in the SUV39H1 overexpressed and control ATDC5 cells. **b** The mRNA level of *suv39h1* in the SUV39H1 overexpressed and control ATDC5 cells. **c** The mRNA levels of *Col2a1* and *Col10a1* in the SUV39H1 overexpressed and control ATDC5 cells. **d** The percentage of EdU-positive cells in the SUV39H1 overexpressed and control ATDC5 cells. CT, empty pcDNA3.1(+) plasmids transfected ATDC5 cells; SUV39H1, pcDNA3.1(+)-SUV39H1 plasmids transfected ATDC5 cells. *n* = 6 independent transfection cells, ^#^*p* < 0.05

**Fig. 6:**
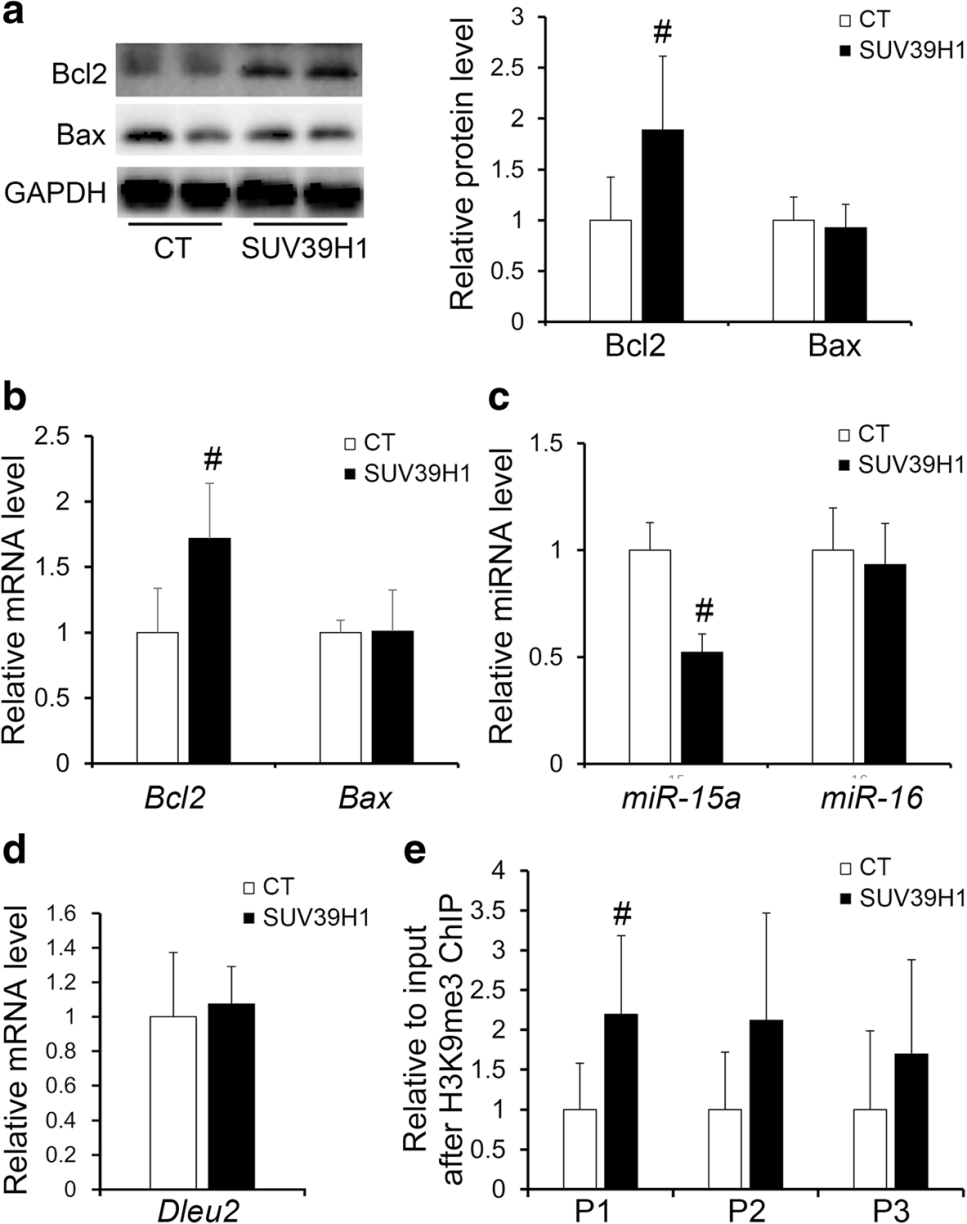
Changes of miR-15a/Bcl2 pathway in SUV39H1-overexpressing ATDC5 cells. **a**, **b** The protein and mRNA levels of Bcl2 and Bax in SUV39H1-overexpressing and control ATDC5 cells, *n* = 6 independent transfection cells. **c** The expression levels of miR-15a and miR-16 in SUV39H1-overexpressing and control ATDC5 cells, *n* = 6 independent transfection cells. **d** The mRNA levels of miR-15a/16 host gene in SUV39H1-overexpressing and control ATDC5 cells, *n* = 6 independent transfection cells. **e** The levels of H3K9me3 in the promoter region of miR-15a/16 host gene in SUV39H1-overexpressing and control ATDC5 cells assessed by ChIP assay, *n* = 3 independent transfection cells. P1––P3, three pairs of primers in the promoter region of miR-15a/16 host gene. ^#^*p* < 0.05

To verify the effect of elevated levels of H3K9me3 and SUV39H1 on chondrocytes, we overexpressed the highly conserved gene SUV39H1 in ATDC5 cells, which is regarded as a promising in vitro model to study the factors that influence cell behaviors during chondrogenesis (Additional file 2: Figure S3) [31]. Successful overexpression of SUV39H1 was observed not only at the mRNA level but also at the protein level (Fig. 5a, b). Additionally, overexpression of SUV39H1 led to an increase in the level of H3K9me3 (Fig. 5a). Similar to the results obtained in facet joint chondrocytes, elevated levels of H3K9me3 and SUV39H1 increased both the mRNA and protein levels of Bcl2 rather than Bax (Fig. 6a, b). Although the mRNA level of *Col10a1* did not change, the mRNA level of *Col2a1* was significantly increased in SUV39H1-overexpressing ATDC5 cells compared with control cells (Fig. 5c). And the 5-ethynyl-2′ -deoxyuridine (EdU) staining results showed increased cell proliferation in SUV39H1-overexpressing ATDC5 cells compared with control cells (Fig. 5d). In addition, the expression level of miR-15a instead of miR-16 was significantly decreased in SUV39H1-overexpressing ATDC5 cells (Fig. 6c). In accordance with the changes observed in IS patients, the H3K9me3 level was also significantly increased in the promoter region of *Dleu2* in SUV39H1-overexpressing ATDC5 cells, although no differences were found in the mRNA levels of *Dleu2* between SUV39H1-overexpressing and control ATDC5 cells (Fig. 6d, e and Additional file 2: Figure S4).

Taken together, these results suggest that H3K9me3 levels elevated by SUV39H1 in the promoter of the miR-15a/miR-16 host gene may decrease the expression of miR-15a inconsistent with its host gene expression, which could further increase the expression of Bcl2. The increased expression of Bcl2 may promote chondrocyte proliferation and ultimately impact spinal growth.

## Discussion

Environmental and lifestyle factors are thought to play an important role in the onset of IS in light of discordant findings for monozygotic twins with AIS [9, 32]. In general, environmental and lifestyle factors could be hormones, nutrition, traffic pollution, noise, alcohol, smoking, viruses, drugs, medications, toxins, physical activity, playing a musical instrument, the type and weight of school bags, the time spent studying, sleep posture, and the number of hours spent sleeping [2, 9, 32]. Although no lifestyle-related factor was identified to be significantly associated with AIS, classical ballet training, a family history of scoliosis, and low BMI may be associated with AIS [32]. Moreover, early exposure to indoor heated swimming pools has been found to increase the independent odds of AIS [33]. Therefore, epigenetics, which is thought to represent the link between environmental factors and phenotypic differences, is a new approach to examine the etiopathogenesis of IS, given that epigenetic differences have arisen during the lifetime of phenotypic discordant monozygotic twins [9, 34].

So far, both DNA methylation and noncoding RNAs have been reported to be connected with IS in some studies [14–17]. However, little research has been conducted on the association between histone modification and IS. As one of the major histone modifications, histone methylation is deeply involved in endochondral ossification and articular cartilage development [23]. Ito et al. found that the histone methylase protein arginine *N*-methyltransferase-4 (PRMT4) could regulate endochondral osteogenesis and chondrocyte proliferation through the methylation of sex-determining region Y-box 9 (Sox9) [35]. Conditional inactivation of H3K27-specific methyltransferase enhancer of zeste homolog 2 (EZH2) prevented craniofacial bone and cartilage formation [36]. H3K9 methyltransferases, such as SUV39H1 and SUV39H2, and H3K9me3 were detected in prehypertrophic and hypertrophic chondrocytes in the growth plate of fetal mice [37]. Czvitkovich et al. observed that overexpression of SUV39H1 induces altered proliferation and differentiation in transgenic mice [38]. In addition, DOT1L, a single nucleotide polymorphism, was reported to be strongly associated with IS and played a role in chondrogenic differentiation and adult articular cartilage [18, 39].

In our study, we examined the tri-methylation levels of H3K4, H3K9, H3K27, H3K36, and H3K79 in facet joint chondrocytes of both IS patients and the control group. Significant upregulation of the level of H3K9me3 was observed, as well as the specific methyltransferase SUV39H1 (Fig. 2). Combining these results with the histopathological findings of the narrowed facet joint cartilage layer but increased number of smaller chondrocytes and expression of *COL2A1* (Fig. 1), we further investigated the proliferation of facet joint chondrocytes. Accordingly, PCNA-positive cells and the expression levels of Bcl2 were increased in IS patients (Fig. 3), which may suggest increased proliferative chondrocytes in the facet joint of IS patients.

Previous studies have revealed faster growth of AIS patients during puberty and overgrowth in the anterior compared to the posterior spinal column in AIS patients, which indicated the presence of abnormal regulation and modulation of skeletal growth and endochondral ossification in patients with AIS, although the underlying mechanism is unclear [4, 40, 41]. Since the inferior facet joint pertains to the posterior column of the spine and there was an increase in proliferative facet joint chondrocytes in the IS patients we observed, the abnormal growth of the facet joint may occur in IS patients. This abnormal growth may further play roles in the development and progression of IS. However, generally, the increased expression levels of H3K9me3 and SUV39H1 cannot upregulate the expression of Bcl2 directly because of its function of gene repression. Surprisingly, the expression of the negative regulator of Bcl2, miR-15a, was significantly decreased in the facet joint chondrocytes of IS patients compared with the control group (Fig. 4). Moreover, the increased H3K9me3 levels in the promoter region of the miR-15a host gene, as evidenced by the ChIP assay, suggested that the downregulation of miR-15a may result from the elevation in H3K9me3 levels and SUV39H1 expression (Fig. 4). In parallel, overexpression of SUV39H1 in ATDC5 cells led to the analogous results that the expression of miR-15a was decreased while the H3K9me3 levels in the promoter region of the miR-15a host gene, the expression levels of Bcl2 and *Col2a1* were increased, and the cell proliferation was also increased (Figs. 5 and 6).

Therefore, our study showed that SUV39H1 and H3K9me3 can regulate chondrocyte proliferation via miR-15a/Bcl2 signaling, although the reason of the inconsistent expression of miR-15a and its host gene *DLEU2* in both IS patients and SUV39H1-overexpressing ATDC5 cells were still unclear. Because studies had shown that intronic miRNA could feedback regulate its host gene, and Morenos et al. had observed that downregulation of *DLEU2* by the increased *DLEU2* promoter DNA methylation in pediatric acute myeloid leukemia was independent of embedded miR-15a/16-1 [42–44]. Thus, we considered that complex mechanisms and processes like feedback regulation, post-transcriptional effects of miRNAs or host genes may be involved, and a large amount of further research may be well needed to figure it out. In addition to chondrocyte proliferation, the progression of endochondral ossification may be related to abnormal spinal growth in IS patients, given the markedly decreased cartilage lacuna and lighter blue color as well as the downregulated expression of *COL10A1* in the facet joint cartilage of IS patients compared with the control group (Fig. 1). Nevertheless, the role of aberrant endochondral ossification in the development and progression of IS and the exact underlying mechanism requires further investigation.

## Conclusions

**Fig. 7:**
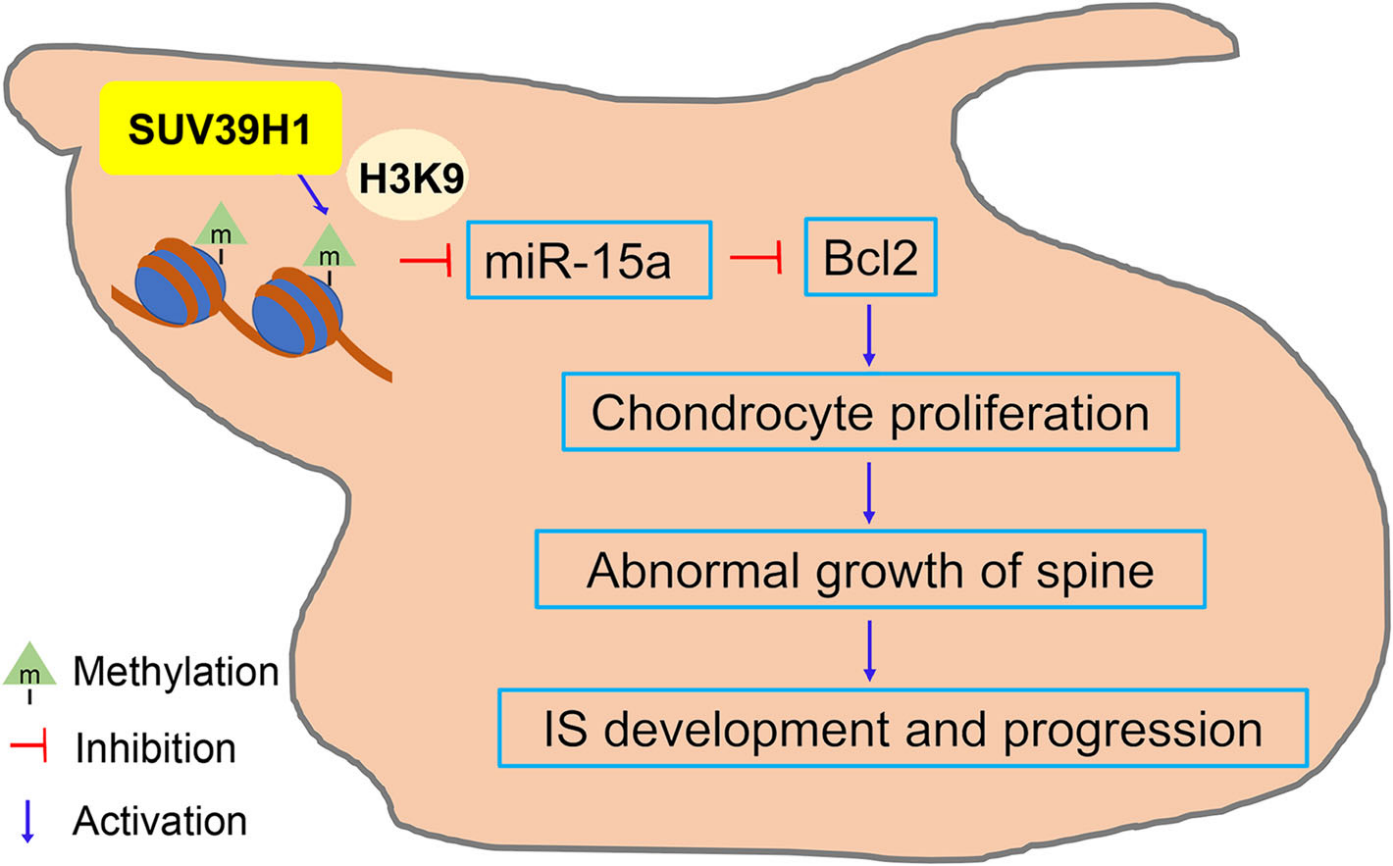
Models for the mechanism of histone H3K9 tri-methylation in the development of IS. Changes of H3K9me3 level may enhance chondrocyte proliferation via miR-15a/Bcl2 pathway, which could lead to abnormal spinal growth and further be associated with the development or progression of IS

In conclusion, as shown in Fig. 7, the increase in H3K9me3, induced by SUV39H1 in chondrocytes of the inferior articular process, activated Bcl2 by inhibiting the expression of miR-15a. However, the dysregulation of the miR-15a/Bcl2 pathway could affect the proliferation of chondrocytes and further lead to abnormal spine growth, which results in the development and progression of IS (Fig. 7). Because of the unclear etiology of IS, prevention-aimed biomechanical intervention rather than effective causality-rooted treatment of IS has been widely applied. Since earlier intervention of IS is a better treatment, further knowledge of the pathogenesis of IS is still urgently needed. In this study, the association of increased H3K9me3 levels at the promoter of miR-15a host gene with the development of IS has been revealed in some way, and it may lay a theoretical foundation for better prevention and treatment of IS in the future.

## Methods

### Patients and controls

The inferior facet joint of 11 IS patients and 10 controls (non-IS patients with lumbar disk herniation and spine fracture), scheduled to be removed during the posterior approach surgery, were collected carefully and harmlessly (Table 1 and Additional file 1: Table S1). The patients and controls were identified based on their clinical manifestations, X-ray, CT, MRI results, etc. The exclusion criteria of the patients and controls were as follows: individuals with severe neuromuscular or genetic disease or routinely using hormones or immune inhibitors.

### Histological analysis and pathological evaluation

Fixed and decalcified facet joint tissues were routinely embedded in paraffin and cut into 5 μm thickness. Facet joint sections were deparaffinized and rehydrated in decreasing concentrations of ethanol and then stained with H&E as previously reported [45]. For analysis, pictures of 3–5 different fields per sample were taken under a Leica microscope equipped with a digital CCD (Leica Camera, Wetzlar, Germany), and semi-quantitative analyses of facet joint cartilage histology were performed in a blinded way.

### Immunofluorescent staining

Deparaffinized and rehydrated facet joint sections or fixed cells were blocked by 5% bovine serum albumin with 0.3% trion X-100 at room temperature (RT) for 30 min and then were incubated with primary antibody for overnight at 4 °C. After extensive washing, sections were incubated with fluorescein-labeled anti-rabbit immunoglobulin G (Servicebio, Wuhan, China) for 2 h at RT. Slides or cells were co-stained with DAPI (Servicebio). The primary antibody against PCNA (Catalogue GB11010-1) and Collagen II (Catalogue GB12021) was purchased from Servicebio. For analysis, pictures of 3–5 different fields per sample were taken under a Leica fluorescence microscope equipped with a digital CCD, and semi-quantitative analyses were performed.

### Extraction of primary chondrocyte and cell culture

The facet joint tissues of IS patients and control group were washed with phosphate buffer solution (PBS) containing 1% penicillin-streptomycin, and then the cartilage layer was carefully dissected and chopped. The chopped cartilage was digested at 37 °C with 0.25% trypsin for 30 min and with 0.1% collagenase II for 4 h successively. The cell pellet obtained after screening and centrifuging were resuspended with the complete culture medium and cultivated in proper dishes. Toluidine blue staining and Collagen II immunofluorescent staining were used for chondrocytes identification (Additional file 2: Figure S5), and the expanded cultured P3 cells were used for subsequent experiments. Primary chondrocytes and ATDC5 cell lines were cultured in high glucose DMEM (Hyclone, Logan, USA) supplemented with 10% fetal bovine serum and 1% penicillin-streptomycin at 37 °C/5% CO_2_.

### EdU staining

To analyze the ATDC5 proliferation after transfection, EdU staining was conducted using the BeyoClick™ EdU Cell Proliferation Kit with Alexa Fluor 594 (Beyotime Biotechnology, Jiangsu, China, Cat. No. C00788S) according to the manufacturer’s instructions. EdU was added into the medium until the final concentration was 10 μM, and the cells were incubated for 2 hours. After the incubation, the cells were washed with PBS to remove the DMEM and the free EdU probe and then fixed in 4% paraformaldehyde at RT for 15 min. After being co-stained with DAPI, the cells were observed under a Leica fluorescence microscope. EdU positive cells were counted at 5 different areas per sample and reported as a percentage of EdU-positive cells.

### Western blotting

Primary chondrocytes were sonicated in RIPA lysis buffer (Beyotime Biotechnology, Jiangsu, China) to obtain whole-cell lysates. Western blot analysis was performed as previously reported and detected by ChemiDoc MP Imager (Bio-Rad Laboratories, Inc., Hercules, CA, USA) [46]. Antibodies for glyceraldehyde-3-phosphate dehydrogenase (GAPDH) (Catalogue 10494-1-AP), Bcl2 (Catalogue 12789-1-AP), and Bax (Catalogue 50599-2-Ig) were obtained from Proteintech Group, Inc, (Chicago, IL, USA), while the remaining antibodies were purchased from Cell Signaling Technologies (Danvers, MA, USA) (Additional file 1: Table S2). The expression levels of target proteins were quantified with the Quantity One 1-D Analysis Software (Bio-Rad) and normalized to the GAPDH in the same sample.

### Real-time PCR

Total cell RNA was extracted using Trizon (KWBio, Beijing, China). For mRNA analysis, equal nanograms of RNA of each sample were reverse transcribed into cDNA using HiFiScript cDNA Synthesis Kit (KWBio). The PCR was performed by NovoStart® Probe qPCR Super Mix (Novoprotein, Shanghai, China) and the transcriptional levels were quantified using GAPDH in the same sample as internal control. For miRNA analysis, equal mass of RNA of each sample was reverse transcribed into cDNA using All-in-One miRNA First-Strand cDNA Synthesis Kit and the real-time PCR was performed with specific primers for the targeted miRNAs using miRNA qPCR Kit (GeneCopoeia, Rockville, MD, USA). The expression levels were quantified using RNU6-B in the same sample as internal control. And the relative fold change was calculated using the 2^−ΔΔCT^. All of the sequences of primers used are described in Tables 2 and 3 and Additional file 1: Table S3.

**Table 2:** Primers sequences for genes

GENE	Primer sequence (5′–3′)	Length (bp)
*H*-*ACAN*	F: ACCGCATCTAATTTGTCCGC	201
	R: AACGATTGCACTGCTCTTGG	
*H*-*COL2A1*	F: CATCCCACCCTCTCACAGTT	166
	R: GGGCATTTGACTCACACCAG	
*H*-*GAPDH*	F: TGACCCCTTCATTGACCTCA	166
	R: ATCGCCCCACTTGATTTTGG	
*H*-*COL10A1*	F: AAGGGAGAAAGAGGACCTGC	180
	R: TGGCCCTGTCTCACCTTTAG	
*H*-*SUV39H1*	F: TTTGCCACAAGAACCATCCG	183
	R: GTATTTGCGGCAGGACTCAG	
*H*-*SUV39H2*	F: TCGTCTTCCCCGAATAGCAT	169
	R: GCAAGTCACAGCTCCACATT	
*H*-*BCL2*	F: CAAGTGTTCCGCGTGATTGA	153
	R: CAGAGGAAAAGCAACGGGG	
*H*-*BAX*	F: GTGCCGGAACTGATCAGAAC	162
	R: CCAAAGTAGGAGAGGAGGCC	
*H*-*DLEU2*	F: GAGACCTCTACTTAGCTCAGCA	232
	R: TGCTATGCCAACTTAACAGGG	
*M*-*Dleu2*	F: TCTCTCGGGCAGAAACCTAC	219
	R: TCGTTTGTGTGCTCTGGAAG	
*M*-*Col2a1*	F: CAACACAATCCATTGCGAAC	159
	R: TCTGCCCAGTTCAGGTCTCT	
*M*-*Col10a1*	F: CAAGCCAGGCTATGGAAGTC	154
	R: AGCTGGGCCAATATCTCCTT	
*M*-*Bcl2*	F: CACACACACACATTCAGGCA	154
	R: GGCAATTCCTGGTTCGGTTT	
*M*-*Bax*	F: GGATGATTGCTGACGTGGAC	175
	R: ATGGTTCTGATCAGCTCGGG	
*M*-*Gapdh*	F: CCCACTCTTCCACCTTCGAT	181
	R: CTTGCTCAGTGTCCTTGCTG	

*Abbreviation*: *F* forward, *R* reverse, *H* human, *M* mouse

**Table 3:** Primers sequences for miRNAs

miRNAs	Primer sequence (5′–3′)	
miR-15a	F: TAGCAGCACATAATGGTTTGTG	
miR-16	F: TAGCAGCACGTAAATATTGGCG	
miR-let7d	F: AGAGGTAGTAGGTTGCATAGTT	
miR-29b	F: TAGCACCATTTGAAATCAGTGTT	
RNU6-B6	F: CGCAAGGATGACACGCAAAT	

*Abbreviation*: *F* forward

### ChIP assay

ChIP experiment was performed as previously reported [45] with some modifications. Briefly, primary chondrocytes or ATDC5 cells were fixed with 1% formaldehyde and stopped by 0.125 M glycine. Pellets were resuspended in WB/IP lysis buffer (Beyotime Biotechnology). DNA was sheared into 200–1000 bp in length, and an aliquot was saved as input. Ten micrograms of DNA from each sample were diluted in WB/IP lysis buffer containing H3K9me3 antibody (Catalogue 13969, CST, Danvers, MA, USA) or IgG (Beyotime Biotechnology) plus salmon sperm DNA-protein A+G agarose (Beyotime Biotechnology) for overnight incubation. The eluted DNA-protein cross-links were reversed by proteinase K incubating at 65 °C. DNA was extracted and PCR analyses were performed on miR-15a host gene *DLEU2* promoter regions. Primer sequences of ChIP-PCR for human and mouse were shown in Tables 4 and 5, respectively, and the location of primers was indicated in Additional file 2: Figure S6. The input samples were used as an internal control for comparison between samples.

**Table 4:** Primers sequences in the promoter region of Human *DLEU2*

Primers	Sequence (5′–3′)	Length (bp)
Primer 1	F: CAAATACGGGTCCTGCTT	195
	R: GTAAAGTTGTTCCGAGGCTT	
Primer 2	F: TTCTCTTGCTTTCCCGAC	270
	R: GCAGGACCCGTATTTGTTTA	
Primer 3	F: TGCCCTTTGCTCCAAGTA	242
	R: AAGAGGCGGTATTGACAGC	

*Abbreviation*: *F* forward, *R* reverse

**Table 5:** Primers sequences in the promoter region of Mouse *Dleu2*

Primers	Sequence (5′–3′)	Length (bp)
Primer 1	F: TAGGCTCGGACAGGTTATCC	227
	R: GGGAGAGGGAGGTAGAAGTT	
Primer 2	F: AAGGCTCTCTACTTCCTCGG	203
	R: CGGTGGCTTGGAGTTTAGT	
Primer 3	F: TAAACTCCAAGCCACCGAG	169
	R: GGTCGTGTGATTGAACCGT	

*Abbreviation*: *F* forward, *R* reverse

### Plasmids and transfection

The CDS sequence of human SUVS9H1 was amplified by Phanta HS Super-Fidelity DNA Polymerase (Vazyme Biotech Co., Nanjing, China), and inserted into the pcDNA3.1+ vector by NheI/BamHI (New England Biolabs Inc., Ipswich, MA, USA) digestion. The sequenced positive plasmids were transfected into ATDC5 cells by lipofectamine 3000 (Invitrogen, Carlsbad, USA) following the user guide. The CDS amplifying primers were listed in Table 6.

**Table 6:** Primers sequences for construction of human SUV39H1- pcDNA3.1(+)

Primers sequence (5′–3′)	CDS length (bp)
F: CGGCTAGCATGGCGGAAAATTTAAAAG	1239
R: CGGGATCCCTAGAAGAGGTATTTGCGG	

*Abbreviation*: *F* forward, *R* reverse, *CDS* coding sequence

### Statistical analysis

All results were expressed as mean ± SD (standard deviation). Statistical significance was determined by analyzing the data of variance by the Student’s *t* test, except for that the gender difference was analyzed by *x*^2^ test. Differences were considered statistically significant when *p* < 0.05.

## Additional files

Additional file 1: **Table S1.** Clinical data of study subjects. **Table S2.** List of antibodies from Cell Signaling Technologies. **Table S3.** Primers sequences for genes. (DOCX 20 kb)

Additional file 2: **Figure S1.** No significant changes of other H3K9 methyltransferases and demethylases between facet joint chondrocytes of IS patients and controls. **Figure S2.** ChIP-PCR data of IS patients and controls. **Figure S3.** SUV39H1 is highly conserved in different species. **Figure S4.** ChIP-PCR data of SUV39H1-overexpressing and control ATDC5 cells. **Figure S5.** Identification of extracted human facet joint chondrocytes. **Figure S6.** Characterization of primers for ChIP-PCR. (DOCX 3899 kb)

## Data Availability

All data generated during this study are included in this published article and its supplementary information files.

## Abbreviations

ACAN: Aggrecan
AIS: Adolescent idiopathic scoliosis
Bax: BCL2 associated x
Bcl2: B-cell lymphoma-2
ChIP: Chromatin immunoprecipitation
COL10A1: Collagen type X
COL2A1: Collagen type II
DLEU2: Deleted in lymphocytic leukemia 2
DOT1L: Disruptor of telomeric silencing 1-like
EdU: 5-ethynyl-2′ -deoxyuridine
EZH2: Enhancer of zeste homolog 2
GAPDH: Glyceraldehyde-3-phosphate dehydrogenase
H&E: Hematoxylin-eosin
H3K79: Histone H3 lysine 79
IS: Idiopathic scoliosis
KDM4A-C: Lysine (K)-specific demethylases 4 A-C
lncRNAs: Long noncoding RNAs
miRNAs: microRNAs
PBS: Phosphate buffer solution
PCNA: Proliferating cell nuclear antigen
PRMT4: Protein arginine N-methyltransferase-4
RT: Room temperature
SD: Standard deviation
SETDB1: SET domain bifurcated-1
Sox9: Sex-determining region Y-Box 9
SUV39H1/2: Suppressor of variegation 3-9, drosophila homolog of 1/2
