# Supplementary material for: Suv39h1 promotes facet joint chondrocyte proliferation by targeting miR-15a/Bcl2 in idiopathic scoliosis patients
Source: Clin Epigenetics. 2019 Jul 23;11:107. doi: 10.1186/s13148-019-0706-1 (PMC6651996; doi:10.1186/s13148-019-0706-1)
Supplement: Supplementary file 1 — Table S1. Clinical data of study subjects. Table S2. List of antibodies from Cell Signaling Technologies. Table S3. Primers sequences for genes. (DOCX 20 kb) [file 13148_2019_706_MOESM1_ESM.docx]

| Group | Age | Sex | Lenke type | Cause of the surgical intervention |
| --- | --- | --- | --- | --- |
| Control 1 | 16 | M | - | Lumbar burst fracture |
| Control 2 | 17 | M | - | Lumbar disc herniation |
| Control 3 | 19 | M | - | Lumbar disc herniation |
| Control 4 | 19 | M | - | Lumbar disc herniation |
| Control 5 | 28 | F | - | Lumbar disc herniation |
| Control 6 | 16 | M | - | Lumbar disc herniation |
| Control 7 | 16 | F | - | Lumbar disc herniation |
| Control 8 | 19 | M | - | Lumbar disc herniation |
| Control 9 | 20 | F | - | Traumatic lumbar disc herniation |
| Control 10 | 28 | F | - | Lumbar burst fracture |
| IS patient 1 | 17 | F | 4 | Scoliosis |
| IS patient 2 | 16 | F | 5 | Scoliosis |
| IS patient 3 | 19 | F | 5 | Scoliosis |
| IS patient 4 | 15 | F | 6 | Scoliosis |
| IS patient 5 | 21 | F | 5 | Scoliosis |
| IS patient 6 | 18 | F | 1 | Scoliosis |
| IS patient 7 | 15 | M | 1 | Scoliosis |
| IS patient 8 | 16 | M | 2 | Scoliosis |
| IS patient 9 | 16 | M | 1 | Scoliosis |
| IS patient 10 | 16 | M | 6 | Scoliosis |
| IS patient 11 | 16 | M | 5 | Scoliosis |

**Table S1. Clinical data of study subjects**

Control 2, control 3 and control 10 were excluded in the Real-time quantitative PCR, Western blotting and ChIP assay because the primary facet joint chondrocytes had not been extracted.

**Table S2. List of antibodies from Cell Signaling Technologies**

| Antibody | Catalogue NO. |
| --- | --- |
| SUV39H1 | 8729 |
| H3K4me3 | 9751 |
| H3K9me3 | 13969 |
| H3K27me3 | 9733 |
| H3K36me3 | 4909 |
| H3K79me3 | 4260 |
| H3 | 4499 |

**Table S3. Primers sequences for genes**

| GENE | Primer sequence (5'–3') | Length (bp) |
| --- | --- | --- |
| *H-SETDB1* | F: CGAATTCTGGGCAAGAAGAG | 161 |
|  | R: TCAGCAGGAGGGTGGTAATC |  |
| *H-KDM4A* | F: ATAGCTCTGTGCGGCAAGTT | 162 |
|  | R: CAGACGCAGGATTCACAGAA |  |
| *H-KDM4B* | F: ACCCTGAGAGCATCACGAGT | 199 |
|  | R: TCCTCCAGGGTGAAGATGTC |  |
| *H-KDM4C* | F: TTCGCAGCTGAGCAAGAGTA | 162 |
|  | R: CTTTCCCTCCGATGTAACGA |  |

Abbreviation: F, Forward; R, Reverse; H, human.
