# Supplementary material for: Suv39h1 promotes facet joint chondrocyte proliferation by targeting miR-15a/Bcl2 in idiopathic scoliosis patients
Source: Clin Epigenetics. 2019 Jul 23;11:107. doi: 10.1186/s13148-019-0706-1 (PMC6651996; doi:10.1186/s13148-019-0706-1)
Supplement: Supplementary file 2 [file 13148_2019_706_MOESM2_ESM.docx]

Additioanal file 2

Figure S1. No significant changes of other H3K9 methyltransferases and demethylases between facet joint chondrocytes of IS patients and controls. 

The mRNA levels of H3K9 methyltransferases SETDB1 (SET domain bifurcated-1) (a), lysine (K)-specific demethylase KDM4A, KDM4B and KDM4C (b-d) in IS patients (n=11) and controls (n=7). 

Figure S2. ChIP-PCR data of IS patients and controls. P1-P3, three pairs of primers in the promoter region of human miR-15a/16 host gene. CT, control group; IS, idiopathic scoliosis.

Figure S3. SUV39H1 is highly conserved in different species. 

Figure S4. ChIP-PCR data of SUV39H1-overexpressing and control ATDC5 cells. P1-P3, three pairs of primers in the promoter region of mouse miR-15a/16 host gene. CT, control cells; SU, SUV39H1-overexpressing cells.

Figure S5. Identification of extracted human facet joint chondrocytes. 

a. Toluidine blue stained facet joint chondrocytes; b. Col2/DAPI co-stained facet joint chondrocytes.

Figure S6. Characterization of primers for ChIP-PCR.

Diagrams of ChIP PCR primers for Human DLEU2 (a) and Mouse Dleu2 (b) gene promoter region. The blue lines with arrowheads indicate DLEU2 gene. The green lines indicate CpG Islands. The Orange lines indicate Regulatory Elements. The Grey lines indicate H3K27Ac modification. The blue lines indicate vertebrate conservation. The red, purple, blue arrowheads indicate ChIP PCR primers. E1, the first base sequence of exon 1. The genome region information obtained from UCSC genome database.
